# Prognostic Value of Leucocyte to High-Density Lipoprotein-Cholesterol Ratios in COVID-19 Patients and the Diabetes Subgroup

**DOI:** 10.3389/fendo.2021.727419

**Published:** 2021-09-13

**Authors:** Yuxiu Wang, Jiaoyue Zhang, Huiqing Li, Wen Kong, Juan Zheng, Yan Li, Qi Wei, Qin Li, Li Yang, Ying Xu, Li Li, Hanyu Wang, Hui Sun, Wenfang Xia, Geng Liu, Xueyu Zhong, Kangli Qiu, Han Wang, Hua Liu, Xiaoli Song, Si Xiong, Yumei Liu, Zhenhai Cui, Lulu Chen, Tianshu Zeng

**Affiliations:** ^1^Department of Endocrinology, Union Hospital, Tongji Medical College, Huazhong University of Science and Technology, Wuhan, China; ^2^Hubei Provincial Clinical Research Center for Diabetes and Metabolic Disorders, Wuhan, China; ^3^Department of Endocrinology, Wuhan Wuchang Hospital, Wuchang Hospital Affiliated to Wuhan University of Science and Technology, Wuhan, China; ^4^Department of Endocrinology, Red Cross Hospital of Wuhan City, Wuhan, China; ^5^Department of Endocrinology, General Hospital of the Yangtze River Shipping, Wuhan, China; ^6^Department of Endocrinology, Hankou Hospital of Wuhan City, Wuhan, China; ^7^Department of Endocrinology, The Fifth Hospital of Wuhan, Wuhan, China

**Keywords:** cohort study, coronavirus, COVID-19, neutrophil to high-density lipoprotein-cholesterol, high-density lipoprotein-cholesterol, prognosis

## Abstract

**Background:**

Blood parameters, such as neutrophil-to-lymphocyte ratio, have been identified as reliable inflammatory markers with diagnostic and predictive value for the coronavirus disease 2019 (COVID-19). However, novel hematological parameters derived from high-density lipoprotein-cholesterol (HDL-C) have rarely been studied as indicators for the risk of poor outcomes in patients with severe acute respiratory syndrome-related coronavirus 2 (SARS-CoV-2) infection. Here, we aimed to assess the prognostic value of these novel biomarkers in COVID-19 patients and the diabetes subgroup.

**Methods:**

We conducted a multicenter retrospective cohort study involving all hospitalized patients with COVID-19 from January to March 2020 in five hospitals in Wuhan, China. Demographics, clinical and laboratory findings, and outcomes were recorded. Neutrophil to HDL-C ratio (NHR), monocyte to HDL-C ratio (MHR), lymphocyte to HDL-C ratio (LHR), and platelet to HDL-C ratio (PHR) were investigated and compared in both the overall population and the subgroup with diabetes. The associations between blood parameters at admission with primary composite end-point events (including mechanical ventilation, admission to the intensive care unit, or death) were analyzed using Cox proportional hazards regression models. Receiver operating characteristic curves were used to compare the utility of different blood parameters.

**Results:**

Of 440 patients with COVID-19, 67 (15.2%) were critically ill. On admission, HDL-C concentration was decreased while NHR was high in patients with critical compared with non-critical COVID-19, and were independently associated with poor outcome as continuous variables in the overall population (HR: 0.213, 95% CI 0.090–0.507; HR: 1.066, 95% CI 1.030–1.103, respectively) after adjusting for confounding factors. Additionally, when HDL-C and NHR were examined as categorical variables, the HRs and 95% CIs for tertile 3 vs. tertile 1 were 0.280 (0.128–0.612) and 4.458 (1.817–10.938), respectively. Similar results were observed in the diabetes subgroup. ROC curves showed that the NHR had good performance in predicting worse outcomes. The cutoff point of the NHR was 5.50. However, the data in our present study could not confirm the possible predictive effect of LHR, MHR, and PHR on COVID-19 severity.

**Conclusion:**

Lower HDL-C concentrations and higher NHR at admission were observed in patients with critical COVID-19 than in those with noncritical COVID-19, and were significantly associated with a poor prognosis in COVID-19 patients as well as in the diabetes subgroup.

## Introduction

The coronavirus disease 2019 (COVID-19) pandemic has become a serious global public health crisis, severely threatening people worldwide. Although most cases are either asymptomatic or result in only mild symptoms, a few patients with COVID-19 rapidly develop acute respiratory distress syndrome, septic shock, multiple organ failure, and die (1). Thus, it is crucial to identify reliable predictors to stratify the risk for patients with severe COVID-19 and enable timely intervention and treatment to improve prognosis.

The role of inflammation in the progression of various viral pneumonitis, including COVID-19, has received increasing attention (2). Accumulating evidence suggests that patients with severe COVID-19 exhibit a hyperinflammatory response and impaired immune function (3). Therefore, circulating biomarkers related to the inflammatory status of patients are good potential predictors of the prognosis of patients with COVID-19. Indeed, the neutrophil-to-lymphocyte ratio has been confirmed as a well-established biomarker for predicting poor prognosis due to COVID-19 (4). However, there is an urgent need to identify novel biomarkers associated with COVID-19 progression.

In addition to reverse cholesterol transport, high-density lipoprotein cholesterol (HDL-C) also displays pleiotropic protective functions, including anti-infectious, anti-inflammatory, antioxidant, and antithrombotic effects (5). During infections or acute conditions, HDL-C levels decrease very rapidly (5, 6). Low concentrations of HDL-C have been used as prognostic markers in patients with sepsis, community-acquired pneumonia and other infections (7–9). In addition, several studies have shown that the degree of reduction in HDL-C and apolipoprotein A-I can predict mortality in COVID-19 patients (10, 11). Besides, new hematological parameters related to HDL-C, including neutrophil to HDL-C ratio (NHR) (12), monocyte to HDL-C ratio (MHR) (13), lymphocyte to HDL-C ratio (LHR) (14) and platelet to HDL-C ratio (PHR) (15), have been advocated as potential new indicators of inflammation in recent studies. These biomarkers have been reported in some lung diseases such as chronic obstructive pulmonary disease (16), stroke-associated pneumonia (17), and pulmonary embolism (18), despite limited studies on newly proposed biomarkers. However, how these serological biomarkers change in the peripheral blood and their clinical value in COVID-19 has not yet been reported.

Diabetes is a major comorbidity of COVID-19 and sufferers are at a markedly higher risk of death after severe acute respiratory syndrome-related coronavirus 2 (SARS-CoV-2) infection (19–21). On the other hand, patients with diabetes have a chronic inflammatory condition and a reduced level of HDL-C (22). Therefore, it is clinically important to examine the influence of inflammatory markers in diabetic patients (19). Hyperglycemia can affect immune function. Conversely, SARS-CoV-2 infection-induced inflammation increases insulin resistance, potentially aggravating the impairment of glucose metabolism (23). Whether these ratios have similar predictive effects on poor outcomes in patients with type 2 diabetes and COVID-19 remains unclear.

Thus, we aimed to investigate and compare the prognostic impacts of NHR, MHR, LHR, and PHR in the entire COVID-19 patient population and in the diabetes subgroup, as well as to explore the most useful diagnostic biomarkers and optimal cutoff values.

## Methods

### Study Design and Participants

In this multicenter, retrospective cohort study, participants with COVID-19 admitted to five hospitals in Wuhan from January 1 to March 17, 2020, were enrolled. All hospitals were designated to treat COVID-19 individuals, including the Department of Endocrinology, the Department of Infectious Disease, and the Department of Oncology of the Union Hospital of Tongji Medical College, Huazhong University of Science and Technology, and the Department of Endocrinology in the following four hospitals: the Fifth Hospital of Wuhan, the Wuhan Wuchang Hospital Affiliated to Wuhan University of Science and Technology, the General Hospital of the Yangtze River Shipping, and the Wuhan Hankou Hospital. These seven departments were temporarily converted into isolation wards for patients with COVID-19. We only had access to the data of the seven departments that the investigators were in charge of; thus, data from other departments in the five hospitals were not available.

COVID-19 was diagnosed according to the Diagnosis and Treatment Scheme for the Novel Coronavirus Pneumonia released by the National Health Commission of China (**Supplementary Table 1**). We only included cases with positive results for SARS-CoV-2 by real-time RT-PCR assay of nasal and pharyngeal swab specimens, or positive serum specific IgM and IgG antibodies. Patients were excluded using the following criteria: (1) previous malignancy; (2) no definitive outcome as they were transferred to another hospital; (3) no hematological or HDL-C data available within 3 days of admission (for one of the following reasons: [a] patients had measured before admission; [b] patients tested for these results 3 days after admission). All patients were followed up until discharge or in-hospital death.

The study was approved by the Ethics Committee of the Union Hospital, Tongji Medical College, Huazhong University of Science and Technology(2020-S180). Informed consent was waived as this retrospective study used only anonymous clinical data.

### Data Collection

We obtained data from the electronic medical records of the relevant departments. The following data were collected: demographics, comorbidities, clinical symptoms and signs, laboratory findings, treatments, and outcomes (discharge or death). Two researchers independently reviewed and verified the data collection forms.

Comorbidities included hypertension, diabetes, coronary heart disease (CHD), chronic lung disease, chronic kidney disease, chronic hepatic disease, cerebrovascular disease, and dyslipidemia, which were diagnosed according to standard criteria. Laboratory results including complete blood count, renal and liver function, lipid profiles, and inflammation markers were recorded within 3 days of admission and before steroid therapy. Medication (e.g., antiviral and antibacterial drugs, systemic corticosteroid, immunoglobulin G, use of statins, and anti-diabetic agents) received during hospitalization were also recorded.

NHR was calculated by dividing neutrophil counts by the HDL-C value. MHR was calculated by dividing monocyte counts by the HDL-C value. LHR was calculated by dividing lymphocyte counts by the HDL-C value. PHR was calculated as the platelet count divided by the HDL-C value.

Patients were discharged when they met the following discharge criteria: (1) body temperature returned to normal, lasting for more than 3 days; (2) respiratory symptoms significantly improved; (3) imaging examinations revealed that acute exudative lesions were significantly improved; and (4) two real-time RT-PCR tests for the presence of SARS-CoV-2 yielded negative results (with two samples of respiratory specimens taken over 24 h apart) (24).

### Study Outcomes

In accordance with previous studies (25, 26), the composite of admission to an intensive care unit (ICU), the use of mechanical ventilation, or death were considered as the primary outcome measure to define critical COVID-19. In contrast, patients discharged without the need for ICU admission or mechanical ventilation were classified as having noncritical COVID-19. The date of disease onset was defined as the day when symptoms suggestive of COVID-19 were first observed. Time (days) was calculated from the symptom onset of COVID-19 to the index date of the composite endpoint of inpatients.

### Statistical Analysis

Data are presented as median and interquartile range (IQR) for continuous variables or as counts and proportions for categorical variables. Comparisons between the groups of non-critical and critical COVID-19 were analyzed using the Mann–Whitney U-test for continuous variables and the chi-squared test or Fisher’s exact test for categorical variables as appropriate. Univariate and multivariate Cox regression analyses were conducted to examine the association between the ratios and primary outcomes. Considering the total number of critical patients (n = 67) in this study and to avoid overfitting in the model, five variables (age, sex, hospital, hypertension, and diabetes) were chosen for multivariable analysis on the basis of previous findings. Regression analysis of the parameters associated with the composite outcome was repeated in a subgroup with diabetes (n = 150). To assess the discrimination ability of each indicator for adverse outcomes, receiver operating characteristic (ROC) curves were calculated, and the optimal cutoff values were determined by maximizing the Youden index. Time to a composite endpoint was investigated using survival analysis by a Kaplan–Meier plot and compared using the log-rank test. All analyses and mapping were performed using IBM SPSS (version 25.0) and GraphPad Prism (version 8.0). Statistical analyses were two-sided, and significance was set at P < 0.05.

## Results

### Baseline Characteristics of Study Samples

**Figure 1:**
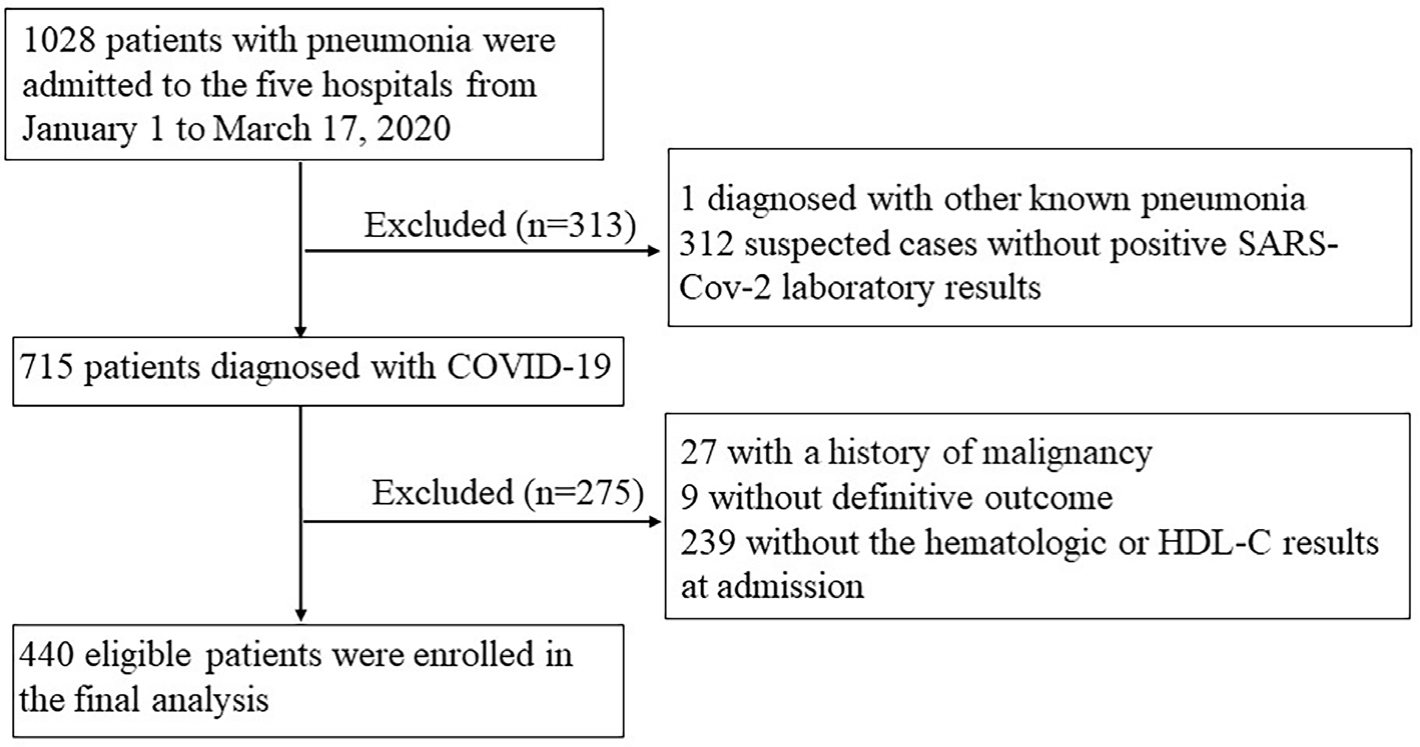
Flow diagram showing the patient selection process.

By March 17, 2020, 1028 patients with pneumonia were admitted to the seven departments across the five hospitals. After excluding one case diagnosed with pneumonia caused by influenza A, 312 suspected cases without positive SARS-Cov-2 laboratory results, 27 with a history of malignancy, 9 without outcome as they were transferred to other hospitals, and 239 patients without blood cell counts or HDL-C data, a total of 440 individuals with COVID-19 were included in the analysis (**Figure 1**). The characteristics of included and excluded patients are presented in **Supplementary Table 2**.

The median age of the participants was 60 years [interquartile range (IQR), 45–68 years], and 215 (48.9%) were men (**Table 1**). The median time from symptom onset to hospital admission was 10 days (range,7–16 days). Dyslipidemia (58.4%) was the most common comorbidity, followed by diabetes (34.1%) and hypertension (30.9%). At admission, the most prevalent symptoms were fever (79.3%), cough (71.6%), and fatigue (48.4%) (**Supplementary Table 3**).

**Table 1:** Demographics and baseline characteristics of patients with COVID-19.

	All Patients (n = 440)	Noncritical COVID-19 (n = 373)	Critical COVID-19 (n = 67)	P value
**Ages**	60 (45-68)	57 (42-67)	69 (60-78)	<0.001
**Sex**				
Female	225 (51.1%)	206 (55.2%)	19 (28.4%)	<0.001
Male	215 (48.9%)	167 (44.8%)	48 (71.6%)	
**Days from illness onset to admission**	10 (7-16)	10 (7-20)	10 (5-12)	0.005
**Comorbidities**				
Hypertension	136 (30.9%)	103 (27.6%)	33 (49.3%)	<0.001
Diabetes	150 (34.1%)	108 (29.0%)	42 (62.7%)	<0.001
Coronary heart disease	44 (10.0%)	34 (9.1%)	10 (14.9%)	0.144
Chronic lung disease	23 (5.2%)	16 (4.3%)	7 (10.4%)	0.074
Chronic liver disease	6 (1.4%)	5 (1.3%)	1 (1.5%)	1
Chronic kidney disease	11 (2.5%)	4 (1.1%)	7 (10.4%)	<0.001
Cerebrovascular disease	21 (4.8%)	11 (2.9%)	10 (14.9%)	<0.001
Dyslipidemia	257 (58.4%)	201 (53.9%)	56 (83.6%)	<0.001
**Treatment**				
Antiviral therapy	397 (90.2%)	336 (90.1%)	61 (91.0%)	0.807
Antibiotic therapy	241 (54.8%)	202 (54.2%)	39 (58.2%)	0.539
Use of corticosteroid	157 (35.8%)	108 (29.0%)	49 (73.1%)	<0.001
Intravenous immunoglobin	122 (27.8%)	86 (231%)	36 (53.7%)	<0.001
Use of statins	45 (10.2%)	33 (8.8%)	12 (17.9%)	0.024

Data are median (IQR) or n (%). P values comparing critical and noncritical COVID-19 are from χ² test, Fisher’s exact test or Mann-Whitney U test.

In this population, 67 (15.2%) had critical COVID-19. Compared with non-critical COVID-19 patients, the critical patients were older, more likely to be male, and more likely to have other comorbidities. Additionally, the critical COVID-19 group was more likely to receive treatment with glucocorticoids [49 (73.1%) *vs*. 108 (29.0%)], intravenous immunoglobulin therapy [36 (53.7%) *vs*. 86 (23.1%)], and statins [12 (17.9%) *vs*. 33 (8.8%)] than the noncritical COVID-19 group (**Table 1**).

### Baseline Laboratory Parameters of Patients With COVID-19

The critical COVID-19 group showed significantly increased levels of neutrophil count, but decreased levels of lymphocyte count, platelet count, as well as multiple lipid profiles, such as total cholesterol (TC), HDL-C, and low-density lipoprotein cholesterol (LDL-C), compared with the non-critical COVID-19 group (P < 0.05). Regarding inflammation indicators, patients with critical COVID-19 had higher levels of NHR and lower levels of LHR than the noncritical group (P < 0.05). No significant differences in monocyte, triglyceride, MHR, and PHR were found (**Table 2**).

**Table 2:** Laboratory findings of patients with COVID-19 on admission to hospital.

	All Patients (n = 440)	Noncritical COVID-19 (n = 373)	Critical COVID-19 (n = 67)	P value
Neutrophils (×10^9^ per L)	3.38 (2.46-4.97)	3.20 (2.37-4.42)	5.94 (3.22-8.47)	<0.001
Lymphocytes (×10^9^ per L)	1.18 (0.78-1.62)	1.26 (0.91-1.71)	0.66 (0.43-0.98)	<0.001
Monocytes (×10^9^ per L)	0.39 (0.29-0.51)	0.40 (0.29-0.52)	0.33 (0.24-0.45)	0.056
Platelets (×109 per L)	192 (144-245)	199 (153-248)	161 (112-233)	<0.001
Total cholesterol (mmol/L)	3.96 (3.38-4.87)	4.02 (3.48-4.92)	3.68 (2.93-4.47)	<0.001
Triglyceride (mmol/L)	1.27 (0.94-1.79)	1.27 (0.95-1.78)	1.33 (0.98-1.88)	0.613
High density lipoprotein cholesterol (mmol/L)	1.04 (0.85-1.30)	1.08 (0.89-1.33)	0.85 (0.71-0.95)	<0.001
Low density lipoprotein cholesterol(mmol/L)	2.30 (1.84-3.00)	2.37 (1.90-3.07)	1.89 (1.56-2.59)	<0.001
Neutrophil to high-density lipoprotein cholesterol ratio (NHR)	3.22 (2.18-5.00)	3.05 (2.02-4.31)	7.71 (4.00-10.96)	<0.001
Monocyte to high-density lipoprotein cholesterol ratio (MHR)	0.38 (0.26-0.51)	0.37 (0.25-0.49)	0.39 (0.27-0.65)	0.118
Lymphocyte to high-density lipoprotein cholesterol ratio (LHR)	1.13 (0.75-1.56)	1.20 (0.81-1.63)	0.82 (0.53-1.11)	<0.001
Platelet to high-density lipoprotein cholesterol ratio (PHR)	175.33 (129.03-250.88)	175.00 (129.36-248.42)	191.74 (115.46-293.83)	0.581

Data are median (IQR). P values comparing critical and noncritical COVID-19 are from Mann-Whitney U test.

### Associations of Biomarkers With Outcome in the Whole COVID-19 Patient Population

**Figure 2:**
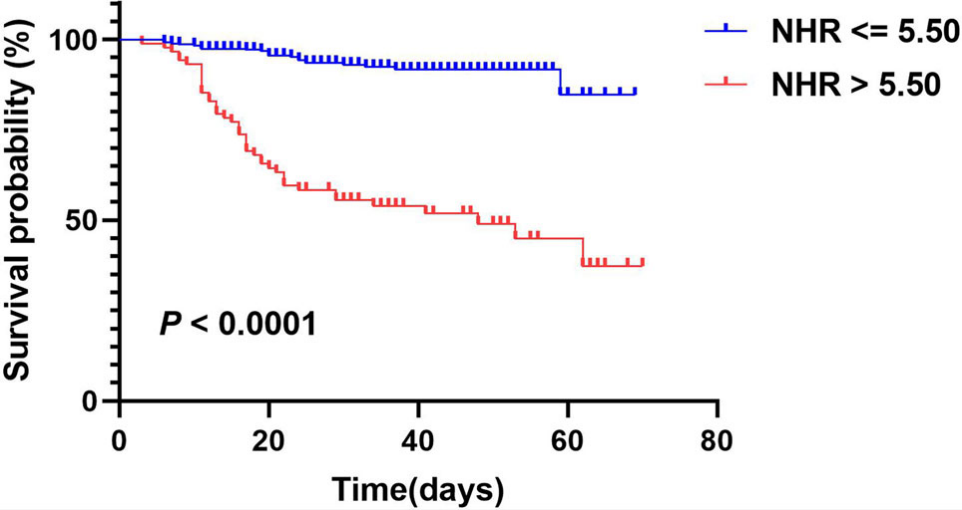
Kaplan-Meier survival curves for primary composite endpoint according to NHR optimal cutoff value. A log-rank test was used to evaluate difference between groups.

According to univariate Cox regression analysis, HDL-C, NHR, and LHR levels were associated with adverse outcomes as both continuous and categorical variables (divided by tertiles). Notably, based on multivariate Cox regression analysis, we found that HDL-C (HR: 0.213, 95% CI: 0.090-0.507, P < 0.001) and NHR (HR: 1.066, 95% CI: 1.030-1.103, P < 0.001) were still independently associated with worse outcomes after adjusting for age, sex, hospital, hypertension, and diabetes (**Table 3**), while the association with LHR was attenuated to insignificance (HR: 0.767, 95% CI: 0.501-1.174). Furthermore, patients with the highest tertile of NHR displayed the highest risk for the primary endpoint, while patients with the highest tertile of HDL-C showed the lowest risk (HR: 4.458, 95% CI: 1.817–10.938; HR: 0.280, 95% CI: 0.128–0.612, respectively). Remarkably, ROC analysis revealed that NHR remained valuable for the primary endpoints, with an AUC > 0.80. At a threshold of 5.50, the AUC of the ROC curve of NHR was 0.81 (95% CI: 0.74–0.87, P < 0.001) (**Table 4**). HDL-C and LHR showed weak discrimination of the critical condition (AUC, 0.72; 95% CI: 0.65–0.79, P < 0.001; AUC: 0.70, 95% CI: 0.63–0.77, P < 0.001, respectively). Moreover, the Kaplan–Meier survival curves and log-rank tests demonstrated that patients with higher NHR (> 5.50) had a higher rate of primary endpoints (divided according to the best threshold) (**Figure 2**).

**Table 3:** Cox proportional hazards regression model for primary composite end point among patients with COVID-19.

Variable	Univariate HR (95% CI)	P value	Adjusted HR* (95% CI)	P value
HDL-C	0.111 (0.047-0.260)	<0.001	0.213 (0.090-0.507)	<0.001
Q1	Ref		Ref	
Q2	0.365 (0.210-0.635)	<0.001	0.495 (0.279-0.879)	0.016
Q3	0.166 (0.078-0.353)	<0.001	0.280 (0.128-0.612)	0.001
NHR	1.082 (1.062-1.102)	<0.001	1.066 (1.030-1.103)	<0.001
Q1	Ref		Ref	
Q2	1.811 (0.679-4.828)	0.235	1.365 (0.496-3.754)	0.547
Q3	7.946 (3.396-18.589)	<0.001	4.458 (1.817-10.938)	0.001
MHR	1.896 (1.208-2.974)	0.005	1.396 (0.879-2.218)	0.157
Q1	Ref		Ref	
Q2	0.688 (0.356-1.330)	0.281	0.759 (0.378-1.523)	0.437
Q3	1.363 (0.776-2.395)	0.281	1.304 (0.696-2.441)	0.407
LHR	0.461 (0.290-0.732)	0.001	0.767 (0.501-1.174)	0.222
Q1	Ref		Ref	
Q2	0.479 (0.281-0.817)	0.007	0.722 (0.409-1.273)	0.26
Q3	0.182 (0.085-0.390)	<0.001	0.331 (0.148-0.743)	0.007
PHR	1.000 (0.998-1.002)	0.862	1.000 (0.998-1.002)	0.789
Q1	Ref		Ref	
Q2	0.993 (0.545-1.811)	0.982	0.850 (0.450-1.606)	0.617
Q3	1.033 (0.574-1.860)	0.913	1.159 (0.629-2.135)	0.636

*Adjusted for age, sex, hospital, hypertension, and diabetes.

**Table 4:** Diagnostic values of serological indicators in assessment adverse outcome of COVID-19.

	AUC (95% CI)	Best threshold	Sensitivity	Specificity	P
HDL-C	0.72 (0.65-0.79)	0.95	0.67	0.73	<0.001
NHR	0.81 (0.75-0.87)	5.50	0.64	0.88	<0.001
MHR	0.56 (0.48-0.64)	0.56	0.34	0.83	0.119
LHR	0.70 (0.63-0.77)	1.03	0.62	0.75	<0.001
PHR	0.52 (0.44-0.60)	292.21	0.25	0.84	0.581

AUC, Area under ROC Curve.

### Associations of Biomarkers With Outcome in the Diabetic Subgroup

In our study, 150 individuals had diabetes. The baseline characteristics of the subjects with diabetes are presented in **Table 5**. Similar results were observed in the diabetes subgroup; the critical illness exhibited higher NHR while exhibiting lower HDL-C and LHR compared with those with noncritical illness, whereas MHR and PHR were comparable between the groups. We further evaluated the predictive performance of several hematological ratios for primary endpoints. Univariate Cox regression analysis showed that HDL-C and NHR were associated with poor outcomes as both continuous and categorical variables (divided by tertiles). Multivariate Cox analysis showed that when correcting for age, sex, and hospital, HDL-C (HR: 0.338, 95% CI: 0.118–0.971, P = 0.044) and NHR (HR: 1.076, 95% CI: 1.028–1.126, P = 0.002) were independent predictors of adverse outcomes (**Table 6**). Among them, 34 (22.7%) patients received metformin therapy and more noncritical patients received metformin compared with critical patients (30.6% *vs*. 2.4%, P < 0.001). Of note, the metformin group had a lower NHR than the non-metformin group, but the difference was not statistically significant (**Supplementary Table 4**).

**Table 5:** Baseline characteristics of diabetic patients with COVID-19 on admission to hospital.

	All Patients (n = 150)	Noncritical COVID-19 (n = 108)	Critical COVID-19 (n = 42)	P value
Ages	64 (57-71)	62 (55-70)	69 (61-75)	0.001
Sex				
Female	67 (44.7%)	54 (50.0%)	13 (31.0%)	0.035
Male	83 (55.3%)	54 (50.0%)	29 (69.0%)	
Days from illness onset to admission	10 (7-15)	10 (7-20)	10 (7-12)	0.126
Metformin	34 (22.7%)	33 (30.6%)	1 (2.4%)	<0.001
Neutrophils (×10^9^ per L)	4.16 (2.82-6.23)	3.58 (2.63-5.14)	6.83 (4.25-9.32)	<0.001
Lymphocytes (×10^9^ per L)	0.97 (0.57-1.33)	1.11 (0.76-1.51)	0.62 (0.35-0.84)	<0.001
Monocytes (×10^9^ per L)	0.36 (0.26-0.51)	0.39 (0.29-0.53)	0.33 (0.21-0.45)	0.112
Platelets (×109 per L)	182 (128-245)	193 (150-264)	152 (103-204)	<0.001
Total cholesterol (mmol/L)	3.83 (3.20-4.66)	3.96 (3.42-4.70)	3.38 (2.92-4.57)	0.02
Triglyceride (mmol/L)	1.42 (1.05-1.90)	1.37 (1.05-1.87)	1.58 (1.04-2.02)	0.734
High density lipoprotein cholesterol (mmol/L)	0.92 (0.79-1.23)	1.03 (0.84-1.27)	0.85 (0.72-0.96)	0.002
Low density lipoprotein cholesterol(mmol/L)	2.20 (1.69-2.78)	2.22 (1.77-2.80)	1.81 (1.44-2.56)	0.062
Neutrophil to high-density lipoprotein cholesterol ratio (NHR)	4.16 (2.73-7.24)	3.66 (2.44-5.07)	8.64 (4.82-12.29)	<0.001
Monocyte to high-density lipoprotein cholesterol ratio (MHR)	0.38 (0.25-0.51)	0.39 (0.25-0.51)	0.38 (0.25-54)	0.844
Lymphocyte to high-density lipoprotein cholesterol ratio (LHR)	0.96 (0.59-1.45)	1.18 (0.73-1.60)	0.73 (0.49-0.99)	<0.001
Platelet to high-density lipoprotein cholesterol ratio (PHR)	186.18 (129.06-262.53)	187.25 (134.44-263.16)	183.20 (113.79-252.57)	0.451

Data are median (IQR) or n (%). P values comparing critical and noncritical COVID-19 are from χ² test and Mann-Whitney U test.

**Table 6:** Cox proportional hazards regression model for primary end point among diabetic patients with COVID-19.

Variable	Univariate HR (95% CI)	P value	Adjusted HR* (95% CI)	P value
HDL-C	0.294 (0.109-0.791)	0.015	0.338 (0.118-0.971)	0.044
Q1	Ref		Ref	
Q2	0.737 (0.375-1.448)	0.376	0.499 (0.233-1.068)	0.073
Q3	0.302 (0.126-0.720)	0.007	0.307 (0.125-0.755)	0.01
NHR	1.061 (1.036-1.086)	<0.001	1.076 (1.028-1.126)	0.002
Q1	Ref		Ref	
Q2	0.631 (0.200-1.993)	0.433	0.641 (0.196-2.090)	0.46
Q3	4.991 (2.177-11.444)	<0.001	3.961 (1.604-9.783)	0.003
MHR	1.372 (0.816-2.306)	0.233	1.266 (0.736-2.180)	0.394
Q1	Ref		Ref	
Q2	0.628 (0.290-1.358)	0.237	0.565 (0.245-1.305)	0.182
Q3	0.811 (0.393-1.677)	0.572	0.810 (0.354-1.854)	0.618
LHR	0.580 (0.331-1.019)	0.058	0.825 (0.540-1.261)	0.375
Q1	Ref		Ref	
Q2	0.604 (0.313-1.166)	0.133	0.625 (0.300-1.301)	0.209
Q3	0.124 (0.042-0.365)	<0.001	0.168 (0.055-0.514)	0.002
PHR	0.999 (0.996-1.001)	0.308	0.999 (0.997-1.002)	0.53
Q1	Ref		Ref	
Q2	0.633 (0.301-1.330)	0.227	0.542 (0.240-1.223)	0.14
Q3	0.664 (0.316-1.395)	0.28	0.804 (0.366-1.768)	0.587

*Adjusted for age, sex, and hospital.

## Discussion

In this multicenter retrospective cohort study, we explored the association between four novel serological indicators and fatal clinical outcomes in patients with COVID-19. Our findings suggested that low HDL-C concentration and high NHR on admission are closely associated with an increased risk of poor clinical outcome not only in all COVID-19 patients but also in the diabetes subgroup. Additionally, ROC curve analysis showed that NHR can effectively forecast the worse outcome of COVID-19.

The COVID-19 outbreak has caused widespread concern, and the progression to severe illness results in high rates of mortality (40%) in patients (27). Hence, it is essential to determine several early markers to make timely assessment as to which cases will likely become clinically severe. The critical cases showed significantly lower HDL-C concentrations than the non-critical cases in this study. Above all, decreased HDL-C concentration was associated with poor prognosis of COVID-19, which was consistent with previous studies (11, 28). More notably, recent studies have focused on the association between HDL-C-related biomarkers and COVID-19. For example, an increased triglyceride to HDL-C ratio indicates a greater risk of worse prognosis in patients with COVID-19 (29). Additionally, in a retrospective cohort study, a high C-reactive protein to HDL-C ratio was independently associated with an increase in mortality and poor prognosis (28). In the present study, we investigated the leucocyte-to-HDL-C ratio, and our results suggested that elevated NHR was associated with poor clinical outcomes after adjusting for confounding factors.

Several factors may contribute to the increased risks of severity related to higher NHR. First, excessive inflammation is an important features of COVID-19 patients. SARS-CoV-2 infection induces a series of immune responses and causes changes in peripheral white blood cells, such as neutrophilia (30). Furthermore, proinflammatory cytokines such as CRP and IL-6 can directly inhibit apolipoprotein synthesis enzyme activity, resulting in decreased ApoA-1 and HDL-C production (5–7, 31). In this context, the cytokine storm described in COVID-19 patients might induce immune-mediated dyslipidemia, leading to reduced HDL. Our study also demonstrated that CRP and IL-6 levels were higher in the critical COVID-19 group and correlated positively with NHR (**Supplementary Tables 3** and **5**). Consequently, NHR may reflect the overactive status of the inflammatory response, which can cause multiple organ damage and death. Second, HDL-C has the ability to inhibit neutrophil activation, attachment, diffusion, and migration (32). However, a large number of activated neutrophils can influence HDL-C composition and function by altering the structure and content of a variety of apolipoproteins (33). Moreover, a recent study in COVID-19 patients also reported altered HDL-C composition and function during severe COVID-19. For example, paraoxonase 1 (PON-1) is less abundant on HDL particles isolated from COVID-19 patients, which may be degraded by elastase released from neutrophil activation during COVID-19 (34). Consequently, a vicious cycle may occur in severe COVID-19 patients, with neutrophil overproduction resulting in deficiency in HDL-C and further neutrophil activation, which is detrimental to the prognosis of COVID‐19. Third, the NHR has been shown to be associated with many diseases, such as metabolic syndrome (12) and cardiovascular disease (35, 36), which are also known risk factors for severe COVID-19 (37–39).

In our study, 257 (58.4%) patients with COVID-19 had dyslipidemia, and the prevalence of dyslipidemia was much higher than that in other studies (5–32.5%) (40–42). One reason may be that the definition of dyslipidemia itself is rather complicated. Statin is one of the drugs most commonly used by patients with dyslipidemia. Statins have favorable anti-inflammatory effects and have been suggested as adjunct therapy for COVID-19 (43). However, our study found that plasma levels of inflammatory biomarkers were not significantly affected by statin therapy (**Supplementary Table 6**). Of note, critical patients were more likely to use statins than non-critical patients (17.9% *vs*. 8.8%). Although it has been reported that statins may increase the risk of SARS-CoV-2 viral entry by inducing angiotensin-converting enzyme 2 (ACE2) expression (44), it is also worth noting that individuals on statins were older and had a greater incidence of chronic diseases (**Supplementary Table 6**), which may explain the higher proportion of statin use in the critical group. Further studies are urgently needed to explore the efficacy of statins on COVID-19 outcomes.

Diabetes is a common comorbidity in patients with COVID-19 and is associated with greater disease severity and higher mortality of COVID-19 (19–21). This may be attributed to the dysregulated immunological status and the exaggerated pro-inflammatory cytokine response, manifesting as higher ratios of lymphopenia and increased levels of neutrophils, serum CRP, and IL-6 in patients with COVID-19 with pre-existing T2D compared to non-diabetic patients (19, 23). In addition, diabetic patients usually exhibit reduced HDL level (22) and impaired HDL function (45). In view of these findings, the role of these new markers in subsets of diabetes is worth exploring. Similarly, higher neutrophil counts but lower HDL-C levels were observed in critical COVID-19 patients compared with non-critical COVID-19 patients in the diabetic population. Therefore, not surprisingly, the NHR tended to be higher in the noncritical group than in the critical group and was associated with an increased risk of adverse outcomes (HR:1.076, 95% CI:1.028-1.126), after adjusting for age, sex, and hospitals. Generally, patients with diabetes show a higher degree of glycation. Glycated HDL showed much lower antiviral activity against SARS-CoV-2 than native HDL (46). In addition, hyperglycemia in diabetes primes neutrophils to release neutrophil extracellular traps (NETs) (47) which might further contribute to the cytokine storm, systemic inflammatory response syndrome (SIRS), and sepsis in COVID-19. Thus, diabetic patients with high NHR are more likely to develop critical illness with COVID-19 because of uncontrolled inflammation. Similar results were also seen in hypertension patients but not CHD patients (**Supplementary Tables 7–10**). This may be due to the limited sample size and the small observed events, which limits the statistical power of this explorative study.

Acute inflammation caused by viral infection may result in dyslipidemia in patients, and lipid metabolism is known to play an important role in the host immune response (5, 6). In fact, inflammation and lipid abnormalities are considered to be associated with poor outcomes in COVID-19. These comprehensive biomarkers might be more reliable and have a better ability to reflect the inflammatory status and lipid metabolism. Furthermore, they are simply calculated from the leukocyte subsets and HDL-C, which are routinely checked, inexpensive, and readily available biomarkers.

Previous studies have independently assessed the clinical significance of these biomarkers in various patients, but only one preprint report so far has investigated the relationship with COVID-19, and only involving MHR (48), which showed a higher value of MHR in male patients than in female patients. Here, we evaluated the early predictive value of NHR, LHR, MHR, and PHR in SARS-Cov-2 infection. To the best of our knowledge, this is the first time that these markers have been simultaneously investigated in COVID-19. Our study will provide a supplement to the research on COVID-19 pneumonia epidemics and references for clinicians to identify individuals at risk.

However, there were several limitations to our study. First, this was a retrospective study and the sample size was relatively small; therefore, further prospective studies with larger cohorts are required to verify our conclusions. Second, the time from the onset of symptoms to the time of serum sample collection when patients were admitted to the hospital varied among patients, which may have caused some bias in the analysis of the relationship between blood biomarkers and COVID-19. Third, a proportion of patients were excluded due to missing data, which may have led to selection bias. Fourth, although we adjusted for several known potential confounders, residual and unmeasured confounding factors might not be fully considered. Finally, the makeup of the study population might limit the generalizability of the results to other ethnic groups.

In conclusion, this retrospective cohort study indicates that lower HDL-C concentration and higher NHR on admission were found in patients with critical COVID-19 than in those without critical COVID-19 and were independently associated with adverse events not only in the overall COVID-19 patients but also in the diabetes subgroup after adjusting for confounding factors. However, further prospective cohort studies are required to confirm our findings.

## Data Availability Statement

The raw data supporting the conclusions of this article will be made available by the authors, without undue reservation.

## Ethics Statement

The studies involving human participants were reviewed and approved by Ethics Committee of the Union Hospital, Tongji Medical College, Huazhong University of Science and Technology. Written informed consent for participation was not required for this study in accordance with the national legislation and the institutional requirements.

## Author Contributions

JiZ, LC, and TZ conceived and supervised the overall study. QW, YX, LL, QL, LY, HYW, GL, XZ, KQ, YaL, HW, YW, XS, HLiu, SX, YuL, and ZC collected the epidemiological and clinical data. HYW, KQ, HW, YW, GL, and XZ summarized all data. YW did the analysis. JiZ and YW drafted the manuscript. HLi, WK, JuZ, WX, and HS contributed to the discussion. LC and TZ revised the final manuscript. TZ is the guarantor of this work and, as such, had full access to all the data in the study and takes responsibility for the integrity of the data and the accuracy of the data analysis. All authors contributed to the article and approved the submitted version.

## Funding

Funding to support this survey was provided by the grant from the National Key Projects of Research and Development of the Ministry of Science and Technology, China (2020YFC0845700), HUST COVID-19 Rapid Response Call (2020kfyXGYJ067) and DMRFP_ II _07 from SHMHDF.

## Conflict of Interest

The authors declare that the research was conducted in the absence of any commercial or financial relationships that could be construed as a potential conflict of interest.

## Publisher’s Note

All claims expressed in this article are solely those of the authors and do not necessarily represent those of their affiliated organizations, or those of the publisher, the editors and the reviewers. Any product that may be evaluated in this article, or claim that may be made by its manufacturer, is not guaranteed or endorsed by the publisher.

## References

[1] Yang X Yu Y Xu J Shu H Xia J Liu H. Clinical Course and Outcomes of Critically Ill Patients With SARS-CoV-2 Pneumonia in Wuhan, China: A Single-Centered, Retrospective, Observational Study. Lancet Respir Med (2020) 8(5):475–81. doi: 10.1016/s2213-2600(20)30079-5 PMC7102538 32105632

[2] Zhu N Zhang D Wang W Li X Yang B Song J. A Novel Coronavirus From Patients With Pneumonia in China, 2019. N Engl J Med (2020) 382(8):727–33. doi: 10.1056/NEJMoa2001017 PMC7092803 31978945

[3] Mehta P McAuley DF Brown M Sanchez E Tattersall RS Manson JJ. COVID-19: Consider Cytokine Storm Syndromes and Immunosuppression. Lancet (2020) 395(10229):1033–4. doi: 10.1016/S0140-6736(20)30628-0 PMC7270045 32192578

[4] Li X Liu C Mao Z Xiao M Wang L Qi S. Predictive Values of Neutrophil-to-Lymphocyte Ratio on Disease Severity and Mortality in COVID-19 Patients: A Systematic Review and Meta-Analysis. Crit Care (London England) (2020) 24(1):647. doi: 10.1186/s13054-020-03374-8 PMC7667659 33198786

[5] Tanaka S Couret D Tran-Dinh A Duranteau J Montravers P Schwendeman A. High-Density Lipoproteins During Sepsis: From Bench to Bedside. Crit Care (London England) (2020) 24(1):134. doi: 10.1186/s13054-020-02860-3 PMC7140566 32264946

[6] Catapano AL Pirillo A Bonacina F Norata GD. HDL in Innate and Adaptive Immunity. Cardiovasc Res (2014) 103(3):372–83. doi: 10.1093/cvr/cvu150 24935428

[7] Pirillo A Catapano AL Norata GD. HDL in Infectious Diseases and Sepsis. Handb Exp Pharmacol (2015) 224:483–508. doi: 10.1007/978-3-319-09665-0_15 25522999

[8] Chien JY Jerng JS Yu CJ Yang PC. Low Serum Level of High-Density Lipoprotein Cholesterol is a Poor Prognostic Factor for Severe Sepsis. Crit Care Med (2005) 33(8):1688–93. doi: 10.1097/01.ccm.0000171183.79525.6b 16096442

[9] Chien YF Chen CY Hsu CL Chen KY Yu CJ. Decreased Serum Level of Lipoprotein Cholesterol is a Poor Prognostic Factor for Patients With Severe Community-Acquired Pneumonia That Required Intensive Care Unit Admission. J Crit Care (2015) 30(3):506–10. doi: 10.1016/j.jcrc.2015.01.001 25702844

[10] Sun JT Chen Z Nie P Ge H Shen L Yang F. Lipid Profile Features and Their Associations With Disease Severity and Mortality in Patients With COVID-19. Front Cardiovasc Med (2020) 7:584987. doi: 10.3389/fcvm.2020.584987 33344516 PMC7746652

[11] Wang G Zhang Q Zhao X Dong H Wu C Wu F. Low High-Density Lipoprotein Level is Correlated With the Severity of COVID-19 Patients: An Observational Study. Lipids Health Dis (2020) 19(1):204. doi: 10.1186/s12944-020-01382-9 32892746 PMC7475024

[12] Chen T Chen H Xiao H Tang H Xiang Z Wang X. Comparison of the Value of Neutrophil to High-Density Lipoprotein Cholesterol Ratio and Lymphocyte to High-Density Lipoprotein Cholesterol Ratio for Predicting Metabolic Syndrome Among a Population in the Southern Coast of China. Diabetes Metab Syndr Obes Targets Ther (2020) 13:597–605. doi: 10.2147/dmso.S238990 PMC7053653 32184639

[13] Uslu AU Sekin Y Tarhan G Canakcı N Gunduz M Karagulle M. Evaluation of Monocyte to High-Density Lipoprotein Cholesterol Ratio in the Presence and Severity of Metabolic Syndrome. Clin Appl Thrombosis/Hemostasis Off J Int Acad Clin Appl Thrombosis/Hemostasis (2018) 24(5):828–33. doi: 10.1177/1076029617741362 PMC6714883 29212375

[14] Chen H Xiong C Shao X Ning J Gao P Xiao H. Lymphocyte To High-Density Lipoprotein Ratio As A New Indicator Of Inflammation And Metabolic Syndrome. Diabetes Metab Syndr Obes Targets Ther (2019) 12:2117–23. doi: 10.2147/dmso.S219363 PMC6798814 31686883

[15] Jialal I Jialal G Adams-Huet B. The Platelet to High Density Lipoprotein -Cholesterol Ratio is a Valid Biomarker of Nascent Metabolic Syndrome. Diabetes/Metabolism Res Rev (2020) 2020:e3403. doi: 10.1002/dmrr.3403 32886844

[16] Huang Y Jiang B Miao X Ma J Wang J Ding K. The Relationship of Lymphocyte to High-Density Lipoprotein Ratio With Pulmonary Function in COPD. Int J Chron Obstruct Pulmon Dis (2020) 15:3159–69. doi: 10.2147/copd.S276372 PMC7718883 33293805

[17] Sun Y Lu J Zheng D Qian J Zhang H Xing D. Predictive Value of Monocyte to HDL Cholesterol Ratio for Stroke-Associated Pneumonia in Patients With Acute Ischemic Stroke. Acta Neurol Belg (2020). doi: 10.1007/s13760-020-01418-y 32638269

[18] Avci A Biricik S Avci BS Yesiloglu O Sumbul HE Icme F. The New Prognostic Factor for Pulmonary Embolism: The Ratio of Monocyte Count to HDL Cholesterol. Am J Emergency Med (2021) 15:212–6. doi: 10.1016/j.ajem.2020.07.026 33071082

[19] Zhu L She ZG Cheng X Qin JJ Zhang XJ Cai J. Association of Blood Glucose Control and Outcomes in Patients With COVID-19 and Pre-Existing Type 2 Diabetes. Cell Metab (2020) 31(6):1068–77.e3. doi: 10.1016/j.cmet.2020.04.021 32369736 PMC7252168

[20] Zhang J Kong W Xia P Xu Y Li L Li Q. Impaired Fasting Glucose and Diabetes Are Related to Higher Risks of Complications and Mortality Among Patients With Coronavirus Disease 2019. Front Endocrinol (Lausanne) (2020) 11:525. doi: 10.3389/fendo.2020.00525 32754119 PMC7365851

[21] Kong W Zhang J Xu Y Li L Li Q Yang L. Letter to the Editor: Fasting Plasma Glucose Associated With Mortality Rate in T2DM Patients With COVID-19 Infection. Metabolism: Clin Exp (2020) 108:154255. doi: 10.1016/j.metabol.2020.154255 PMC7189865 32360210

[22] Wu L Parhofer KG. Diabetic Dyslipidemia. Metabolism: Clin Exp (2014) 63(12):1469–79. doi: 10.1016/j.metabol.2014.08.010 25242435

[23] Lim S Bae JH Kwon HS Nauck MA. COVID-19 and Diabetes Mellitus: From Pathophysiology to Clinical Management. Nat Rev Endocrinol (2021) 17(1):11–30. doi: 10.1038/s41574-020-00435-4 33188364 PMC7664589

[24] China. NHCotPsRo. Diagnosis and Treatment Plan of Coronavirus Disease 2019 (COVID-19) (Trial Version 6) (2020) (Accessed 2 March 2021).

[25] Guan WJ Ni ZY Hu Y Liang WH Ou CQ He JX. Clinical Characteristics of Coronavirus Disease 2019 in China. N Engl J Med (2020) 382(18):1708–20. doi: 10.1056/NEJMoa2002032 PMC7092819 32109013

[26] Sardu C D'Onofrio N Balestrieri ML Barbieri M Rizzo MR Messina V. Outcomes in Patients With Hyperglycemia Affected by COVID-19: Can We Do More on Glycemic Control? Diabetes Care (2020) 43(7):1408–15. doi: 10.2337/dc20-0723 PMC7305003 32430456

[27] Wiersinga WJ Rhodes A Cheng AC Peacock SJ Prescott HC. Pathophysiology, Transmission, Diagnosis, and Treatment of Coronavirus Disease 2019 (COVID-19): A Review. JAMA (2020) 324(8):782–93. doi: 10.1001/jama.2020.12839 32648899

[28] Li Y Zhang Y Lu R Dai M Shen M Zhang J. Lipid Metabolism Changes in Patients With Severe COVID-19. Clin Chim Acta (2021) 517:66–73. doi: 10.1016/j.cca.2021.02.011 33639119 PMC7903909

[29] Zhang B Dong C Li S Song X Wei W Liu L. Triglyceride to High-Density Lipoprotein Cholesterol Ratio is an Important Determinant of Cardiovascular Risk and Poor Prognosis in Coronavirus Disease-19: A Retrospective Case Series Study. Diabetes Metab Syndrome Obesity: Targets Ther (2020) 13:3925–36. doi: 10.2147/dmso.S268992 PMC7591232 33122929

[30] Yang L Liu S Liu J Zhang Z Wan X Huang B. COVID-19: Immunopathogenesis and Immunotherapeutics. Signal Trans Targeted Ther (2020) 5(1):128. doi: 10.1038/s41392-020-00243-2 PMC7381863 32712629

[31] Golucci A Marson FAL Ribeiro AF Nogueira RJN. Lipid Profile Associated With the Systemic Inflammatory Response Syndrome and Sepsis in Critically Ill Patients. Nutrition (2018) 55-56:7–14. doi: 10.1016/j.nut.2018.04.007 29960160

[32] Murphy AJ Woollard KJ Suhartoyo A Stirzaker RA Shaw J Sviridov D. Neutrophil Activation is Attenuated by High-Density Lipoprotein and Apolipoprotein A-I in *In Vitro* and *In Vivo* Models of Inflammation. Arteriosclers Thromb Vasc Biol (2011) 31(6):1333–41. doi: 10.1161/atvbaha.111.226258 21474825

[33] Bergt C Marsche G Panzenboeck U Heinecke JW Malle E Sattler W. Human Neutrophils Employ the Myeloperoxidase/Hydrogen Peroxide/Chloride System to Oxidatively Damage Apolipoprotein A-I. Eur J Biochem (2001) 268(12):3523–31. doi: 10.1046/j.1432-1327.2001.02253.x 11422382

[34] Begue F Tanaka S Mouktadi Z Rondeau P Veeren B Diotel N. Altered High-Density Lipoprotein Composition and Functions During Severe COVID-19. Sci Rep (2021) 11(1):2291. doi: 10.1038/s41598-021-81638-1 33504824 PMC7841145

[35] Kou T Luo H Yin L. Relationship Between Neutrophils to HDL-C Ratio and Severity of Coronary Stenosis. BMC Cardiovasc Disord (2021) 21(1):127. doi: 10.1186/s12872-020-01771-z 33676400 PMC7936429

[36] Huang JB Chen YS Ji HY Xie WM Jiang J Ran LS. Neutrophil to High-Density Lipoprotein Ratio has a Superior Prognostic Value in Elderly Patients With Acute Myocardial Infarction: A Comparison Study. Lipids Health disease (2020) 19(1):59. doi: 10.1186/s12944-020-01238-2 PMC7126405 32247314

[37] Xie J Zu Y Alkhatib A Pham TT Gill F Jang A. Metabolic Syndrome and COVID-19 Mortality Among Adult Black Patients in New Orleans. Diabetes Care (2020) 44(1):188–93. doi: 10.2337/dc20-1714 PMC7783937 32843337

[38] Docherty AB Harrison EM Green CA Hardwick HE Pius R Norman L. Features of 20 133 UK Patients in Hospital With Covid-19 Using the ISARIC WHO Clinical Characterisation Protocol: Prospective Observational Cohort Study. Bmj (2020) 369:m1985. doi: 10.1136/bmj.m1985 32444460 PMC7243036

[39] Leung JM Niikura M Yang CWT Sin DD. COVID-19 and COPD. Eur Respir J (2020) 56(2):2002108. doi: 10.1183/13993003.02108-2020 32817205 PMC7424116

[40] Zhang JJ Dong X Cao YY Yuan YD Yang YB Yan YQ. Clinical Characteristics of 140 Patients Infected With SARS-CoV-2 in Wuhan, China. Allergy (2020) 75(7):1730–41. doi: 10.1111/all.14238 32077115

[41] Petrilli CM Jones SA Yang J Rajagopalan H O'Donnell L Chernyak Y. Factors Associated With Hospital Admission and Critical Illness Among 5279 People With Coronavirus Disease 2019 in New York City: Prospective Cohort Study. Bmj (2020) 369:m1966. doi: 10.1136/bmj.m1966 32444366 PMC7243801

[42] Simonnet A Chetboun M Poissy J Raverdy V Noulette J Duhamel A. High Prevalence of Obesity in Severe Acute Respiratory Syndrome Coronavirus-2 (SARS-CoV-2) Requiring Invasive Mechanical Ventilation. Obes (Silver Spring) (2020) 28(7):1195–9. doi: 10.1002/oby.22831 PMC7262326 32271993

[43] Zhang XJ Qin JJ Cheng X Shen L Zhao YC Yuan Y. In-Hospital Use of Statins Is Associated With a Reduced Risk of Mortality Among Individuals With COVID-19. Cell Metab (2020) 32(2):176–87.e4. doi: 10.1016/j.cmet.2020.06.015 32592657 PMC7311917

[44] Shrestha S. Statin Drug Therapy may Increase COVID-19 Infection. Nepalese Med J (2020) 3(2):e0228637. doi: 10.1371/journal.pone.0228637

[45] Morgantini C Natali A Boldrini B Imaizumi S Navab M Fogelman AM. Anti-Inflammatory and Antioxidant Properties of HDLs are Impaired in Type 2 Diabetes. Diabetes (2011) 60(10):2617–23. doi: 10.2337/db11-0378 PMC3178289 21852676

[46] Cho KH Kim JR Lee IC Kwon HJ. Native High-Density Lipoproteins (HDL) With Higher Paraoxonase Exerts a Potent Antiviral Effect Against SARS-CoV-2 (COVID-19), While Glycated HDL Lost the Antiviral Activity. Antioxidants (Basel) (2021) 10(2):209. doi: 10.3390/antiox10020209 33535459 PMC7912765

[47] Wong SL Demers M Martinod K Gallant M Wang Y Goldfine AB. Diabetes Primes Neutrophils to Undergo NETosis, Which Impairs Wound Healing. Nat Med (2015) 21(7):815–9. doi: 10.1038/nm.3887 PMC4631120 26076037

[48] Xingzhong Hu DC Wu L He G Ye W. Low Serum Cholesterol Level Among Patients With COVID-19 Infection in Wenzhou, China (2020). Available at: https://ssrn.com/abstract=3544826.

