# Supplementary material for: Prognostic Value of Leucocyte to High-Density Lipoprotein-Cholesterol Ratios in COVID-19 Patients and the Diabetes Subgroup
Source: Front Endocrinol (Lausanne). 2021 Sep 13;12:727419. doi: 10.3389/fendo.2021.727419 (PMC8473871; doi:10.3389/fendo.2021.727419)
Supplement: Supplementary file 1 [file DataSheet_1.docx]

**Supplementary appendix**

**METHODS**

**Definitions of COVID-19 diagnosis**

Case definition for COVID-19 was divided into suspected case and confirmed case based on the epidemiological history, clinical features and laboratory examination by the National Health Commission of the People’s Republic of China (1). Only confirmed cases were analyzed in our study.

**Table 1：Case Definition for Surveillance of Coronavirus Disease 2019 (COVID-19) by Chinese Health Commission**

| Suspected case  Present at least two of the following conditions of  i. Fever and/or respiratory symptoms (eg, cough, myalgia, fatigue)  ii. Imaging features of viral pneumonia  iii. Normal or low white blood cell count or reduced lymphocyte in early onset  AND  One or more of the following exposures during the 14 days prior to onset of symptoms  1). Travel or residence history in Wuhan, other areas with recent local transmission of COVID-19, or the local community with confirmed patient  2). Close contact* with patient with laboratory-confirmed COVID-19 (positive for nucleic acid test)  3). Close contact with people from Wuhan or surrounding areas or local communities with fever or respiratory symptoms case report  4). Cluster onset (two or more cases of fever and/or respiratory symptoms occurred within 2 weeks in a small area such as home, office, school and class).  Patients without exposure history should meet all of conditions i, ii and iii. |
| --- |
| **Clinically diagnosed case (added in the trial fifth edition but deleted in the trial sixth and seventh edition)**  The suspected case with typical imaging findings of pneumonia (only for Hubei) |
| Confirmed case  Suspected cases have at least one of the following etiological evidence  i. Positive real-time reverse-transcriptase polymerase chain reaction of the patient’s respiratory or blood specimen for COVID-19 nucleic acid  ii. Viral gene sequences in respiratory or blood specimen are highly homologous to COVID-19  iii. Positive serum specific IgM and IgG antibody for COVID-19; The serum specific IgG antibody for COVID-19 changed from negative to positive or the recovery period was 4 times or more higher than the acute period. |

*Close contact is defined as healthcare-related exposures, including direct care for patients with confirmed COVID-19, collaboration with healthcare workers with confirmed COVID-19, visiting or staying in the same closed environment with patients with confirmed COVID-19, or members who live in the same family environment with patients with confirmed COVID-19.

| **Table 2 Baseline clinical characteristics and laboratory findings of included and excluded patients with COVID-19** | | | | |
| --- | --- | --- | --- | --- |
|  | **All Patients (n=1028)** | **Excluded patients (n=588)** | **Included patients (n=440)** | **P value** |
| **Ages** | 60 (47-67) | 60 (48-66) | 60 (45-68) | 0.258 |
| **Sex** |  |  |  |  |
| Female | 524 (51.0%) | 299 (50.9%) | 225 (51.1%) | 0.928 |
| Male | 504 (49.0%) | 289 (49.1%) | 215 (48.9%) |  |
| **Days from illness onset to admission** | 10 (6-15) | 9 (6-13) | 10 (7-16) | <0.001 |
| **Comorbidities** |  |  |  |  |
| Hypertension | 320 (31.1%) | 184 (31.3%) | 136 (30.9%) | 0.895 |
| Diabetes | 220 (21.4%) | 127 (21.7%) | 93 (21.1%) | 0.836 |
| Coronary heart disease | 90 (8.8%) | 46 (7.8%) | 44 (10.0%) | 0.225 |
| Chronic lung disease | 43 (4.2%) | 20 (3.4%) | 23 (5.3%) | 0.145 |
| Chronic liver disease | 24 (2.3%) | 18 (3.1%) | 6 (1.4%) | 0.075 |
| Chronic kidney disease | 20 (1.9%) | 9 (1.5%) | 11 (2.5%) | 0.267 |
| Cerebrovascular disease | 68 (6.6%) | 34 (3.3%) | 34 (3.3%) | 0.214 |
| **Clinical outcome** |  |  |  |  |
| Primary end events | 155 (15.2%) | 84 (14.5%) | 71 (16.1%) | 0.459 |
| Admission to ICU | 35 (3.4%) | 13 (2.2%) | 22 (5.0%) | 0.015 |
| Mechanical ventilation | 103 (10.0%) | 50 (8.5%) | 53 (12.0%) | 0.061 |
| Dead | 112 (11.0%) | 65 (11.2%) | 47 (10.7%) | 0.783 |
| **Laboratory examination** | |  |  |  |
| Leucocytes (×10^9^ per L) | 5.28 (3.94-6.98) | 6.53 (4.82-9.68) | 7.01 (5.36-9.10) | 0.272 |
| Neutrophils (×10^9^ per L) | 3.48 (2.43-5.12) | 3.49 (2.39-5.32) | 3.32 (2.45-4.96) | 0.54 |
| Lymphocytes (×10^9^ per L) | 1.09 (0.72-1.50) | 0.90 (0.69-1.32) | 1.18 (0.78-1.66) | <0.001 |
| Monocytes (×10^9^ per L) | 0.38 (0.27-0.51) | 0.34 (0.25-0.51) | 0.39 (0.28-0.52) | 0.043 |
| Platelets (×109 per L) | 191 (144-247) | 187 (141-252) | 193 (145-245) | 0.765 |
| Alanine aminotransferase(U/L) | 27 (17-42) | 23 (16-41) | 27 (18-43) | 0.57 |
| Aspartate aminotransferase(U/L) | 29 (21-42) | 29 (22-45) | 26 (20-49) | 0.003 |
| Albumin (g/L) | 35.40 (31.60-39.40) | 35.60 (32.20-39.70) | 35.90 (31.80-40.20) | 0.075 |
| Blood urea nitrogen(mmol/L) | 4.40 (3.40-5.75) | 4.30 (3.34-5.66) | 4.62 (3.51-5.88) | 0.114 |
| Serum creatinine (μmol/L) | 66.90 (56.00-81.00) | 68.05 (55.48-83.00) | 67.40 (56.80-81.90) | 0.185 |
| Fasting plasma glucose(mmol/L) | 5.90 (5.16-7.50) | 6.01 (5.25-7.63) | 5.80 (5.12-7.22) | 0.111 |
| Total cholesterol (mmol/L) | 3.86 (3.32-4.66) | 3.79 (3.28-4.39) | 3.97 (3.42-4.87) | <0.001 |
| Triglyceride (mmol/L) | 1.21 (0.93-1.67) | 1.10 (0.88-1.49) | 1.27 (0.95-1.79) | 0.001 |
| High density lipoprotein cholesterol (mmol/L) | 1.02 (0.83-1.27) | 1.00 (0.80-1.23) | 1.04 (0.84-1.31) | 0.027 |
| Low density lipoprotein cholesterol(mmol/L) | 2.30 (1.81-2.91) | 2.29 (1.78-2.79) | 2.31 (1.85-3.01) | 0.226 |
| C-creative protein(mg/L) | 18.40 (3.74-54.50) | 37.35 (5.19-68.20) | 4.60 (3.14-29.08) | <0.001 |
| Lactate dehydrogenase(U/L) | 237 (183-321) | 304 (204-382) | 206 (170-280) | <0.001 |
| Interleukin-6(pg/L） | 8.54 (4.01-20.99) | 14.37 (5.79-27.71) | 6.29 (3.54-13.92) | <0.001 |
| Neutrophil to high-density lipoprotein cholesterol ratio (NHR) | 3.30 (2.17-5.01) | 3.45 (2.14-5.16) | 3.22 (2.18-5.00) | 0.265 |
| Monocyte to high-density lipoprotein cholesterol ratio (MHR) | 0.36 (0.25-0.51) | 0.34 (0.23-0.56) | 0.37 (0.26-0.50) | 0.648 |
| Lymphocyte to high-density lipoprotein cholesterol ratio (LHR) | 1.09 (0.71-1.50) | 0.98 (0.61-1.42) | 1.12 (0.75-1.56) | 0.008 |
| Platelet to high density lipoprotein cholesterol ratio (PHR) | 178.26 (130.04-258.00) | 185.53 (135.33-263.38) | 175.91 (128.88-252.57) | 0.303 |
| Primary end events including admission to ICU, mechanical ventilation and dead. Data are median (IQR) or n (%). P values comparing critical and noncritical COVID-19 are from χ² test, Fisher’s exact test and Mann-Whitney U test. ICU= intensive care unit. | | | | |

| **Table 3 Baseline and laboratory characteristics of patients with COVID-19** | | | | |
| --- | --- | --- | --- | --- |
|  | **All Patients (n=440)** | **Noncritical COVID-19 (n=373)** | **Critical COVID-19 (n=67)** | **P value** |
| **Signs and symptoms at admission** | |  |  |  |
| Fever | 349 (79.3%) | 292 (78.3%) | 57 (85.1%) | 0.206 |
| Fatigue | 213 (48.4%) | 186 (49.9%) | 27 (40.3%) | 0.149 |
| Cough | 315 (71.6%) | 266 (71.3%) | 49 (73.1%) | 0.761 |
| Myalgia | 104 (23.6%) | 91 (24.4%) | 13 (19.4%) | 0.376 |
| Dyspnea | 168 (38.2%) | 124 (33.2%) | 44 (65.7%) | <0.001 |
| Headache | 36 (8.2%) | 33 (8.8%) | 3 (4.5%) | 0.23 |
| Chest pain | 105 (23.9%) | 82 (22.0%) | 23 (34.3%) | 0.029 |
| Diarrhoea | 97 (22.0%) | 83 (22.3%) | 14 (20.9%) | 0.805 |
| Nausea and vomiting | 47 (10.7%) | 40 (10.7%) | 7 (10.4%) | 0.946 |
| Palpitation | 36 (8.2%) | 30 (8.0%) | 6 (9.0%) | 0.802 |
| Anorexia | 159 (36.1%) | 131 (35.1%) | 28 (41.8%) | 0.295 |
| Lipid-lowering agents | 45 (12.6%) | 33 (11.3%) | 12 (18.5%) | 0.113 |
| **Laboratory test** |  |  |  |  |
| Leucocytes (×10^9^ per L) | 5.36 (4.05-7.01) | 5.20 (3.94-6.67) | 7.02 (4.76-9.45) | <0.001 |
| Alanine aminotransferase(U/L) | 27 (18-42) | 27 (17-42) | 32 (19-47) | 0.186 |
| Aspartate aminotransferase(U/L) | 27 (20-40) | 25 (20-36) | 44 (31-60) | <0.001 |
| Albumin (g/L) | 35.80 (31.70-40.10) | 36.85 (32.80-40.50) | 30.70 (26.60-34.45) | <0.001 |
| Blood urea nitrogen(mmol/L) | 4.63 (3.50-5.90) | 4.37 (3.39-5.61) | 6.05 (4.52-9.36) | <0.001 |
| Serum creatinine (μmol/L) | 67.35 (56.68-81.90) | 66.15 (55.83-79.90) | 74.70 (64.60-106.00) | <0.001 |
| Fasting plasma glucose(mmol/L) | 5.82 (5.12-7.27) | 5.62 (5.04-6.82) | 7.48 (6.01-9.52) | <0.001 |
| C-creative protein(mg/L) | 10.55 (3.14-43.50) | 4.29 (3.14-18.55) | 94.00 (36.49-157.00) | <0.001 |
| Lactate dehydrogenase(U/L) | 221 (176-305) | 197 (169-258) | 516 (321-682) | <0.001 |
| Interleukin-6(pg/L） | 6.37 (3.61-13.46) | 5.95 (3.45-11.50) | 91.40 (28.81-161.65) | <0.001 |
| Data are median (IQR) or n (%). P values comparing critical and noncritical COVID-19 are from χ² test, Fisher’s exact test or Mann-Whitney U test. | | | | |

| **Table 4. Laboratory findings of diabetic patients with COVID-19 on admission to hospital** | | | | |
| --- | --- | --- | --- | --- |
|  | **All Patients (n=150)** | **Non-metformin (n=116)** | **Metformin (n=34)** | **P value** |
| Neutrophils (×10^9^ per L) | 4.16 (2.82-6.23) | 4.35 (2.88-6.79) | 3.89 (2.63 -5.45) | 0.353 |
| Lymphocytes (×10^9^ per L) | 0.97 (0.57-1.33) | 0.82 (0.53-1.17） | 1.31 (1.04-1.90) | <0.001 |
| Monocytes (×10^9^ per L) | 0.36 (0.26-0.51) | 0.35 (0.25-0.49) | 0.42 (0.31-0.55) | 0.029 |
| Platelets (×109 per L) | 182 (128-245) | 177 (127-236) | 214 (152-275) | 0.024 |
| Total cholesterol (mmol/L) | 3.83 (3.20-4.66) | 3.73 (3.17-4.57) | 4.27 (3.60-4.99) | 0.021 |
| Triglyceride (mmol/L) | 1.42 (1.05-1.90) | 1.32 (0.99-1.81) | 1.70 (1.24-2.73) | 0.009 |
| High density lipoprotein cholesterol (mmol/L) | 0.92 (0.79-1.23) | 0.90 (0.79-1.22) | 1.03 (0.84-1.27) | 0.256 |
| Low density lipoprotein cholesterol(mmol/L) | 2.20 (1.69-2.78) | 2.03 (1.65-2.72) | 2.42 (1.89-2.84) | 0.076 |
| Neutrophil to high-density lipoprotein cholesterol ratio (NHR) | 4.16 (2.73-7.24) | 4.25 (2.75-8.06) | 3.29 (2.63-5.75) | 0.126 |
| Monocyte to high-density lipoprotein cholesterol ratio (MHR) | 0.38 (0.25-0.51) | 0.38 (0.23-0.51) | 0.40 (0.31-0.51) | 0.339 |
| Lymphocyte to high-density lipoprotein cholesterol ratio (LHR) | 0.96 (0.59-1.45) | 0.84 (0.52-1.27) | 1.34 (1.00-1.84) | 0.332 |
| Platelet to high-density lipoprotein cholesterol ratio (PHR) | 186.18 (129.06-262.53) | 185.88 (121.98-251.79) | 192.84 (143.92-279.04) | <0.001 |
| Data are median (IQR). P values comparing critical and noncritical COVID-19 are from Mann-Whitney U test. | | | | |

| **Table 5. Spearman rank correlations of biomarkers with inflammatory factors** | | | | |
| --- | --- | --- | --- | --- |
|  | CRP | | IL-6 | |
|  | Rho coefficient | P values | Rho coefficient | P values |
| HDL-C | -0.33 | <0.001 | -0.095 | 0.257 |
| NHR | 0.334 | <0.001 | 0.225 | 0.007 |
| MHR | -0.007 | 0.878 | 0.277 | 0.001 |
| LHR | -0.383 | <0.001 | -0.335 | <0.001 |
| PHR | 0.007 | 0.88 | 0.099 | 0.241 |

| **Table 6 Baseline and laboratory findings of COVID-19 patients with statins or without statins on admission to hospital** | | | | |
| --- | --- | --- | --- | --- |
|  | **All Patients (n=440)** | **Non-statins (n=395)** | **Statins (n=45)** | **P value** |
| Ages | 60 (45-68) | 60 (44-75) | 64 (56-74) | 0.003 |
| Hypertension | 136 (30.9%) | 111 (28.1%) | 25 (55.6%) | <0.001 |
| Diabetes | 150 (34.1%) | 131 (33.2%) | 19 (42.2%) | 0.225 |
| Coronary heart disease | 44 (10.0%) | 21 (5.3%) | 23 (51.1%) | <0.001 |
| Chronic lung disease | 23 (5.3%) | 21 (5.3%) | 2 (4.7%) | 1 |
| Chronic liver disease | 6 (1.4%) | 6 (1.5%) | 0 | 1 |
| Chronic kidney disease | 11 (2.5%) | 7 (1.8%) | 4 (8.9%) | 0.017 |
| Cerebrovascular disease | 21 (4.8%) | 13 (3.3%) | 8 (17.8%) | <0.001 |
| Dyslipidemia | 257 (58.4%) | 225 (57.0%) | 32 (71.1%) | 0.068 |
| Neutrophils (×10^9^ per L) | 3.38 (2.46-4.97) | 3.31 (2.38-4.64) | 6.04 (3.68-9.32) | 0.089 |
| Lymphocytes (×10^9^ per L) | 1.18 (0.78-1.62) | 1.40 (0.96-1.85) | 0.73 (0.52-1.40) | 0.839 |
| Monocytes (×10^9^ per L) | 0.39 (0.29-0.51) | 0.40 (0.31-0.56) | 0.41 (0.19-0.52) | 0.767 |
| Platelets (×109 per L) | 192 (144-245) | 208 (161-259) | 177 (118-273) | 0.997 |
| Total cholesterol (mmol/L) | 3.96 (3.38-4.87) | 4.25 (3.62-4.91) | 4.81 (3.44-5.17) | 0.984 |
| Triglyceride (mmol/L) | 1.27 (0.94-1.89) | 1.33 (0.92-1.76) | 1.11 (0.99-2.14) | 0.367 |
| High density lipoprotein cholesterol (mmol/L) | 1.04 (0.85-1.30) | 1.17 (0.95-1.38) | 0.77 (0.74-1.33) | 0.127 |
| Low density lipoprotein cholesterol(mmol/L) | 2.30 (1.84-3.00) | 2.46 (2.01-3.08) | 2.59 (1.87-3.51) | 0.401 |
| Neutrophil to high-density lipoprotein cholesterol ratio (NHR) | 3.22 (2.18-5.00) | 3.18 (2.13-4.92) | 3.67 (2.79-6.28) | 0.069 |
| Monocyte to high-density lipoprotein cholesterol ratio (MHR) | 0.37 (0.26-0.51) | 0.37 (0.26-0.49) | 0.38 (0.25-0.66) | 0.722 |
| Lymphocyte to high-density lipoprotein cholesterol ratio (LHR) | 1.13 (0.75-1.56) | 1.12 (0.75-1.55) | 1.19 (0.80-1.75) | 0.419 |
| Platelet to high-density lipoprotein cholesterol ratio (PHR) | 175.33 (129.03-250.88) | 175 (129-249) | 193 (124-300) | 0.536 |
| Data are median (IQR) or n (%). P values comparing critical and noncritical COVID-19 are from χ² test, Fisher’s exact test or Mann-Whitney U test. | | | | |

| **Table 7. Baseline characteristics of COVID-19 patients with hypertension on admission** | | | | | | | | | | | |  |  |
| --- | --- | --- | --- | --- | --- | --- | --- | --- | --- | --- | --- | --- | --- |
|  | | **All Patients (n=136)** | | | **Noncritical COVID-19 (n=103)** | **Critical COVID-19 (n=33)** | | | | **P value** | |  |  |
| Ages | | 67(60-74) | | | 65 (57-70) | 74 (65-81) | | | | <0.001 | |  |  |
| Sex | |  | | |  |  | | | |  | |  |  |
| Female | | 61 (44.9%) | | | 54 (52.4%) | 7 (21.2%) | | | | 0.002 | |  |  |
| Male | | 75 (55.1%) | | | 49 (47.6%) | 26 (78.8%) | | | |  | |  |  |
| Days from illness onset to admission | | 10 (6-15) | | | 10 (7-20) | 8 (6-10) | | | | 0.008 | |  |  |
| Neutrophils (×10^9^ per L) | | 3.77 (3.04-5.28) | | | 3.65 (2.91-4.60) | 5.96 (3.12-8.33) | | | | 0.002 | |  |  |
| Lymphocytes (×10^9^ per L) | | 1.01 (0.72-1.50) | | | 1.17 (0.83-1.63) | 0.64 (0.43-0.82) | | | | <0.001 | |  |  |
| Monocytes (×10^9^ per L) | | 0.40 (0.27-0.56) | | | 0.42 (0.29-0.58) | 0.33 (0.21-0.44) | | | | 0.021 | |  |  |
| Platelets (×109 per L) | | 194 (142-254) | | | 213 (160-269) | 135 (94-177) | | | | <0.001 | |  |  |
| Total cholesterol (mmol/L) | | 3.91 (3.33-4.87) | | | 4.00 (3.46-4.98) | 3.75 (2.85-4.09) | | | | 0.002 | |  |  |
| Triglyceride (mmol/L) | | 1.41 (0.99-1.90) | | | 1.41 (1.05-1.91) | 1.48 (0.89-1.81) | | | | 0.701 | |  |  |
| High density lipoprotein cholesterol (mmol/L) | | 0.95 (0.79-1.30) | | | 1.05 (0.84-1.37) | 0.82 (0.71-0.90) | | | | <0.001 | |  |  |
| Low density lipoprotein cholesterol(mmol/L) | | 2.29 (1.77-2.85) | | | 2.41 (1.84-3.09) | 1.87 (1.58-2.48) | | | | 0.006 | |  |  |
| Neutrophil to high-density lipoprotein cholesterol ratio (NHR) | | 3.88 (2.50-6.36) | | | 3.46 (2.38-4.78) | 8.30 (3.65-12.00) | | | | <0.001 | |  |  |
| Monocyte to high-density lipoprotein cholesterol ratio (MHR) | | 0.40 (0.27-0.55) | | | 0.40 (0.27-0.53) | 0.39 (0.25-0.65) | | | | 0.692 | |  |  |
| Lymphocyte to high-density lipoprotein cholesterol ratio (LHR) | | 1.07 (0.74-1.47) | | | 1.19 (0.84-1.56) | 0.81 (0.55-1.02) | | | | <0.001 | |  |  |
| Platelet to high-density lipoprotein cholesterol ratio (PHR) | | 191.00 (133.13-263.16) | | | 192 (137-272) | 189 (105-244) | | | | 0.277 | |  |  |
| Data are median (IQR) or n (%). P values comparing critical and noncritical COVID-19 are from χ² test and Mann-Whitney U test. | | | | | | | | | | | |  |  |
| **Table 8. Cox proportional hazards regression model for primary end point among patients with hypertension** | | | | | | | |  |  |  |  |  |  |
| Variable | Univariate HR (95% CI) | | P | Adjusted HR* (95% CI) | | | P |  |  |  |  |  |  |
| HDL-C | 0.096 (0.027-0.347) | | <0.001 | 0.163 (0.043-0.620) | | | 0.008 |  |  |  |  |  |  |
| Q1 | Ref | |  | Ref | | |  |  |  |  |  |  |  |
| Q2 | 0.595 (0.285-1.240) | | 0.166 | 0.758 (0.344-1.669) | | | 0.491 |  |  |  |  |  |  |
| Q3 | 0.137 (0.040-0.465) | | 0.001 | 0.136 (0.036-0.517) | | | 0.003 |  |  |  |  |  |  |
| NHR | 1.062 (1.037-1.087) | | <0.001 | 1.100 (1.030-1.175) | | | 0.004 |  |  |  |  |  |  |
| Q1 | Ref | |  | Ref | | |  |  |  |  |  |  |  |
| Q2 | 0.766 (0.234-2.512) | | 0.66 | 0.461 (0.128-1.662) | | | 0.236 |  |  |  |  |  |  |
| Q3 | 4.111 (1.665-10.150) | | 0.002 | 2.881 (1.070-7.759) | | | 0.036 |  |  |  |  |  |  |
| MHR | 1.795 (0.980-3.288) | | 0.058 | 0.907 (0.363-2.267) | | | 0.835 |  |  |  |  |  |  |
| Q1 | Ref | |  | Ref | | |  |  |  |  |  |  |  |
| Q2 | 0.772 (0.325-1.834) | | 0.558 | 1.024 (0.395-2.651) | | | 0.961 |  |  |  |  |  |  |
| Q3 | 0.891 (0.400-1.985) | | 0.778 | 0.825 (0.319-2.132) | | | 0.691 |  |  |  |  |  |  |
| LHR | 0.267 (0.124-0.577) | | 0.001 | 0.298 (0.131-0.682) | | | 0.004 |  |  |  |  |  |  |
| Q1 | Ref | |  | Ref | | |  |  |  |  |  |  |  |
| Q2 | 0.414 (0.192-0.894) | | 0.025 | 0.491 (0.222-1.086) | | | 0.079 |  |  |  |  |  |  |
| Q3 | 0.166 (0.056-0.490) | | 0.001 | 0143 (0.039-0.530) | | | 0.004 |  |  |  |  |  |  |
| PHR | 0.998 (0.994-1.001) | | 0.259 | 0.999 (0.996-1.002) | | | 0.533 |  |  |  |  |  |  |
| Q1 | Ref | |  | Ref | | |  |  |  |  |  |  |  |
| Q2 | 0.936 (0.424-2.064) | | 0.87 | 0.951 (0.387-2.338) | | | 0.912 |  |  |  |  |  |  |
| Q3 | 0.594 (0.243-1.455) | | 0.254 | (0.673-0.265-1.712) | | | 0.406 |  |  |  |  |  |  |
| *Adjusted for age, sex, and hospital. | | | | | | | |  |  | |  | |  |

| **Table 9. Baseline characteristics of COVID-19 patients with coronary heart disease on admission** | | | | |
| --- | --- | --- | --- | --- |
|  | **All Patients (n=44)** | **Noncritical COVID-19 (n=34)** | **Critical COVID-19 (n=10)** | **P value** |
| Ages | 68 (62-77) | 67 (60-74) | 72 (65-82) | 0.127 |
| Sex |  |  |  |  |
| Female | 17 (38.6%) | 14 (41.2%) | 3 (30.0%) | 0.788 |
| Male | 27 (61.4%) | 20 (58.8%) | 7 (70.0%) |  |
| Days from illness onset to admission | 10 (5-15) | 11 (6-19) | 9 (5-11) | 0.133 |
| Neutrophils (×10^9^ per L) | 3.71 (2.93-5.26) | 3.71 (2.96-5.00) | 4.45 (2.77-7.01) | 0.484 |
| Lymphocytes (×10^9^ per L) | 1.01 (0.61-1.40) | 1.18 (0.79-1.55) | 0.58 (0.42-1.01) | 0.004 |
| Monocytes (×10^9^ per L) | 0.35 (0.21-0.58) | 0.42 (0.31-0.58) | 0.22 (0.16-0.43) | 0.077 |
| Platelets (×109 per L) | 178 (112-218) | 189 (136-239) | 110 (91-159) | 0.003 |
| Total cholesterol (mmol/L) | 3.70 (3.15-4.77) | 3.77 (3.21-4.92) | 3.21 (2.70-3.98) | 0.075 |
| Triglyceride (mmol/L) | 1.22 (1.00-1.61) | 1.25 (1.08-2.05) | 1.01 (0.86-1.58) | 0.084 |
| High density lipoprotein cholesterol (mmol/L) | 0.93 (0.80-1.31) | 0.94 (0.83-1.33) | 0.94 (0.78-1.13) | 0.695 |
| Low density lipoprotein cholesterol(mmol/L) | 1.94 (1.37-2.64) | 2.03 (1.45-2.69) | 1.71 (1.31-2.40) | 0.275 |
| Neutrophil to high-density lipoprotein cholesterol ratio (NHR) | 4.01 (2.60-5.37) | 3.96 (2.67-5.04) | 5.09 (2.47-8.17) | 0.251 |
| Monocyte to high-density lipoprotein cholesterol ratio (MHR) | 0.39 (0.20-0.54) | 0.42 (0.28-0.55) | 0.23 (0.18-0.53) | 0.153 |
| Lymphocyte to high-density lipoprotein cholesterol ratio (LHR) | 1.04 (0.64-1.30) | 1.11 (0.81-1.59) | 0.61 (0.40-1.08) | 0.025 |
| Platelet to high-density lipoprotein cholesterol ratio (PHR) | 177.92 (102.83-247.37) | 193.28 (123.58-257.22) | 115.18 (71.81-202.36) | 0.038 |
| Data are median (IQR) or n (%). P values comparing critical and noncritical COVID-19 are from χ² test and Mann-Whitney U test. | | | | |

| **Table 10. Cox proportional hazards regression model for primary end point among patients with** **coronary heart disease** | | | | |
| --- | --- | --- | --- | --- |
| Variable | Univariate HR (95% CI) | P | Adjusted HR* (95% CI) | P |
| HDL-C | 0.486 (0.065-3.604) | 0.48 | 0.436 (0.047-4.049) | 0.465 |
| Q1 | Ref |  | Ref |  |
| Q2 | 1.527 (0.364-6.408) | 0.563 | 1.159 (0.242-5.558) | 0.853 |
| Q3 | 0.656 (0.109-3.948) | 0.645 | 0.223 (0.017-2.852) | 0.249 |
| NHR | 1.186 (0.974-1.444) | 0.09 | 1.510 (1.113-2.047) | 0.008 |
| Q1 | Ref |  | Ref |  |
| Q2 | 0.231 (0.026-2.068) | 0.19 | 0.041 (0.002-0.804) | 0.035 |
| Q3 | 1.314 (0.352-4.905) | 0.685 | 2.538 (0.338-19.029) | 0.365 |
| MHR | 0.245 (0.013-4.600) | 0.347 | 0.325 (0.011-9.522) | 0.514 |
| Q1 | Ref |  | Ref |  |
| Q2 | 0.174 (0.021-1.448) | 0.106 | 0.092 (0.006-1.362) | 0.083 |
| Q3 | 0.521 (0.130-2.085) | 0.357 | 0.329 (0.055-1.969) | 0.223 |
| LHR | 0.172 (0.037-0.804） | 0.025 | 0.101 (0.010-0.999) | 0.05 |
| Q1 | Ref |  | Ref |  |
| Q2 | 0.383 (0.094-1.564) | 0.181 | 0.351 (0.066-1.865) | 0.219 |
| Q3 | 0.145 (0.017-1.231) | 0.077 | 0.100 (0.009-1.121) | 0.062 |
| PHR | 0.992 (0.984-1.000） | 0.04 | 0.990 (0.980-1.000) | 0.053 |
| Q1 | Ref |  | Ref |  |
| Q2 | 0.085 (0.010-0.719) | 0.024 | 0.032 (0.002-0.426) | 0.009 |
| Q3 | 0.225 (0.046-1.094) | 0.064 | 0.146 (0.022-0.968) | 0.046 |
| *Adjusted for age, sex, and hospital. | | | | |
